# Analyzing perceptions of a global event using CNN-LSTM deep learning approach: the case of Hajj 1442 (2021)

**DOI:** 10.7717/peerj-cs.1087

**Published:** 2022-09-20

**Authors:** Mohd Khaled Shambour

**Affiliations:** The Custodian of the Two Holy Mosques Institute for Hajj and Umrah Research, Umm Al-Qura University, Makkah, Saudi Arabia

**Keywords:** Hajj rituals, Sentiment analysis, Convolutional Neural Networks (CNN), Long short term memory, Deep learning

## Abstract

Hajj (pilgrimage) is a unique social and religious event in which many Muslims worldwide come to perform Hajj. More than two million people travel to Makkah, Saudi Arabia annually to perform various Hajj rituals for four to five days. However, given the recent outbreak of the coronavirus (COVID-19) and its variants, Hajj in the last 2 years 2020–2021 has been different because pilgrims were limited down to a few thousand to control and prevent the spread of COVID-19. This study employs a deep learning approach to investigate the impressions of pilgrims and others from within and outside the Makkah community during the 1442 AH Hajj season. Approximately 4,300 Hajj-related posts and interactions were collected from social media channels, such as Twitter and YouTube, during the Hajj season Dhul-Hijjah 1–13, 1442 (July 11–23, 2021). Convolutional neural networks (CNNs) and long short-term memory (LSTM) deep learning methods were utilized to investigate people’s impressions from the collected data. The CNN-LSTM approach showed superior performance results compared with other widely used classification models in terms of F-score and accuracy. Findings revealed significantly positive sentiment rates for tweets collected from Mina and Arafa holy sites, with ratios exceeding 4 out of 5. Furthermore, the sentiment analysis (SA) rates for tweets about Hajj and pilgrims varied during the days of Hajj. Some were classified as positive tweets, such as describing joy at receiving the days of Hajj, and some were negative tweets, such as expressing the impression about the hot weather and the level of satisfaction for some services. Moreover, the SA of comments on several YouTube videos revealed positive classified comments, including praise and supplications, and negative classified comments, such as expressing regret that the Hajj was limited to a small number of pilgrims.

## Introduction

In late 2019, the world witnessed the spread of the coronavirus (COVID-19), which infected many industries and companies globally, including the social, health, economic, and educational sectors (Shambour & Abu-Hashem, 2021; Singh, Jakhar & Pandey, 2021). The epidemic forced many countries to implement restrictions to combat the transmission of infection and put preventive measures to push the public into social distancing, which in turn had a substantial influence on many aspects and activities of life that people are familiar with (Mittal, Mittal & Aggarwal, 2021).

Among those aspects is the practice of numerous Islamic religious rites, such as mosque prayers, Hajj and Umrah rituals, and Eid (Islamic festival) (Alam et al., 2021). Despite the COVID-19 outbreak, the Saudi government was keen to continue Hajj rituals in the Islamic lunar calendar 1441–1442 (AH), 2020–2021 (AD) but limited the number of pilgrims from within the country and ordering strict health controls (Jokhdar et al., 2021). The reduction of pilgrims greatly impacted the Islamic society, particularly the Makkah society, because people in Makkah used to provide pilgrims with housing, accommodation, and transportation. This study attempts to utilize convolutional neural networks (CNN) and long short-term memory (LSTM) deep learning approach to reveal the perceptions, feelings, and thoughts of pilgrims and individuals from inside and outside Makkah, in which various limitations were enforced during the Hajj season 1442 AH because of the spread of COVID-19.

One of the techniques used to learn about people’s feelings was to use social media, where individuals can share their thoughts, opinions, and feelings about events and activities that occur in their communities and throughout the world (Janjua et al., 2021; Shambour & Gutub, 2022). Recently, studies have shown a growing interest in knowing the feelings of users of different social media platforms through machine learning (ML) and lexicon-based algorithms that analyze and categorize what users think across social media platforms, such as Facebook, Twitter, and YouTube. The analysis of such data helps in understanding the thoughts of individuals and society in response to specific events and predicting future trends, such as those related to the COVID-19 pandemic (Mittal, Mittal & Aggarwal, 2021), tourism (Chaabani, Toujani & Akaichi, 2018), disasters (Zaki et al., 2018), and economy (Urlam, 2021). Despite many publications describing several subjects related to tracking and sentiment analytics in the literature, the Arabic literature on sentiment analysis (SA) topics has been comparatively weak compared with that in English (Abu Farha & Magdy, 2021).

In the present study, two of the most popular social media networks, Twitter and YouTube (Statista, 2022), were used to collect more than 11,000 tweets and comments in Arabic on topics strongly related to Hajj events from July 11 to 23, 2021 (1–13 Dhul-Hijjah, the 12th month of the Islamic calendar). CNN-LSTM methods were applied to the acquired data because of their effectiveness in finding good results in SA in Arabic compared with other methods in the literature (Abu Farha & Magdy, 2021). The results were extracted and debated in light of the impressions of pilgrims and individuals from inside and outside the Makkah community. The SA results were linked to the events accompanying the Hajj days.

The rest of the article is organized as follows: “Related literature” presents some relevant studies in the literature. “Methodology” and “Evaluation of the CNN-LSTM model” present the research methodology and an evaluation of the CNN-LSTM model, respectively. “Results and discussion” provides the results and discussions. Finally, “Conclusion and future work” gives the conclusion and future work.

## Related literature

Many users express their feelings and perceptions regarding a variety of issues and themes that they encounter in their everyday lives on social media platforms (Bataineh & Shambour, 2019), which are the most prevalent and commonly used by members of societies with various demographic features. Many businesses and agencies of various organizations have conducted social media user sentiment analyses to improve services or develop future strategies and policies (Singh, Jakhar & Pandey, 2021). The following sub-sections present the applications of CNN-LSTM deep learning approach and studies that looked at the usage of social media in general and in relation to Hajj in particular.

### Applications of the CNN-LSTM deep learning approach

Bekkar et al. (2021) applied a CNN-LSTM deep learning approach to predict air pollution in Beijing. The authors used a dataset of air pollution and air quality concentrations with 13 variables from 12 sites. CNN was used to extract the spatial and internal characteristics of distinct variables. LSTM was utilized to extract the time features and stable prediction results. Results show the superiority of the proposed model compared with other deep learning approaches in the literature. Ombabi, Ouarda & Alimi (2020) employed the CNN-LSTM model for Arabic SA. One-layer CNN architecture was used to extract local features, whereas two-layer LSTM architecture was used to maintain long-term dependencies. Three benchmark datasets were used to validate the proposed method, including large-scale Arabic book reviews (LABR), Arabic sentiment tweets dataset (ASTD), and Arabic sentiment analysis Twitter dataset (ASATD). Authors have reported good performance results for the proposed approach compared with other deep learning approaches, including CNN and neural words embedding, TF-IDF with SVM classifier, and FastText with SVC and Logistic Regression classifiers.

Gill et al. (2022) and Vasumathi (2021) proposed other deep learning applications for fruit image classification. They used various classification models, including CNN, RNN, LSTM, SVM, and fuzzy inference systems, to classify fruit. They reported that the CNN-LSTM model performed better than other classification methods. CNN was used for feature extraction. LSTM was utilized to determine the class based on extracted features.

Umer et al. (2020) proposed an automated news detection method using the CNN-LSTM model. The principal component analysis extracted features from the dataset. Then, using CNN, more beneficial features were extracted, and only those with high relevance values, as determined by the max-pooling layer, were sent to the LSTM layer for sequence modeling. Authors reported that their approach outperformed state-of-the-art approaches, including BERT (Devlin et al., 2019), XLNet (Yang et al., 2019), and RoBERTa (Liu et al., 2019), in terms of accuracy. Numerous studies have used the CNN-LSTM technique to solve various problems in a variety of domains, including wind speed forecast (Chen et al., 2021), facial expression recognition (Hung & Tien, 2021), emotion detection (Abdullah, Hadzikadicy & Shaikhz, 2019), hate speech detection (Duwairi, Hayajneh & Quwaider, 2021), and spam detection (Ghourabi, Mahmood & Alzubi, 2020).

### Sentiment analysis and its applications

Bollen, Mao & Zeng (2011) proposed a mechanism for investigating stock market expectations based on daily Twitter updates of the general mood. The proposed method assessed the user’s feelings and moods, which were then used for further time series analysis to visualize the state of the stock market. Pang, Lee & Vaithyanathan (2002) investigated how well several ML classifiers performed in movie reviews. They employed several unigram- and bigram-based feature extractors. In training data, polarity signals were also employed as star ratings.

Furthermore, Alayba et al. (2017) presented an Arabic dataset for public opinion on health services based on Twitter. The authors used various SA techniques, including naive Bayes (NB), CNNs, and support vector machine (SVM), to test the presented dataset. Baker et al. (2020) and Ilyas & Alowibdi (2018) developed methods for detecting diseases based on ML algorithms. The authors collected tweets from Arab countries using several topics related to the disease. The collected tweets were then categorized, filtered, and analyzed. According to the authors, the proposed methods performed well in tracking the spread of some infectious diseases in the region.

Bati (2015) and Elgamal (2016) highlighted the potential of using big data techniques to collect, analyze, and visualize data of Twitter users in Hajj. Feelings and impressions about the services were provided. The results were then used to support decision-making in creating actions to expand and improve the services provided. The authors discussed a framework for collecting Hajj-related tweets and the tools for analyzing them in general steps but did not practically apply them in their studies.

Zahrani, Khaldi & Qahtani (2015) collected almost five million Arabic and English tweets during the 2016 Hajj season. They classified the tweets into three categories: positive, neutral, and negative. The authors also categorized tweets into spatial and temporal categories based on Hajj-related terms. The analysis revealed a wide range of sentiments among tweeters in response to events during the Hajj. Although the authors acquired many Hajj-related tweets, the study did not explain the classification method or the steps of SA and its implementation.

Ottom & Nahar (2021) investigated the accuracy of many ML and lexical techniques in identifying and analyzing 3,175 English tweets during the 2018 Hajj season. The authors applied three ML approaches, namely, SVM, K-nearest neighbor, and NB. For the lexicon-based approach, they used the text analyzer Blobanalyzer. The researchers noted that ML approaches provided better results than the lexical approach in terms of accuracy but did not delve into how the results related to the Hajj events.

### Discussion

From the precedence that a limited number of studies have addressed the SA of social media users during Hajj, the analysis and interpretation of users’ posts have weaknesses, and the studies did not provide a clear mechanism for analyzing users’ feelings and linking the results to various occurrences during Hajj days.

Furthermore, previous Hajj-related studies have not covered the mechanism for assessing sentiments in Arabic, providing further impetus to focus on establishing a mechanism for analyzing and interpreting users’ feelings for one of the most human gatherings in the world. Finally, this study examines the opinions of pilgrims and individuals from inside and outside the Makkah community on a variety of topics relating to Hajj, particularly in light of the expansion of the COVID-19 pandemic, which has resulted in a decrease in the number of pilgrims and confines them to residents in Saudi Arabia for 2020 and 2021.

## Methodology

The research methodology comprises four stages. The first stage is to review the literature on tracking and evaluating individuals’ impressions and sentiments in communities. The second stage is data collecting, which involves using Twitter and YouTube to collect data from pilgrims and individuals inside and outside the Makkah community. The third stage is data analysis, in which appropriate analysis processes, such as data preparation, text representation, feature extraction, and SA, are used. Finally, the results are presented and discussed in the last stage. The following is an additional explanation for each stage.

### Reviewing literature

A critical review of previous studies was carried out to reveal previous work done to address and overcome similar problems and discover the research gap. The previous section included an overview of the literature on tracking and evaluating users’ thoughts and feelings on various subjects and applications.

### Data collection

User posts on Twitter and YouTube platforms were collected from pilgrims and individuals from inside and outside the Makkah community between 1 and 13 Dhul-Hijjah 1442 AH, July 11 to 23, 2021. A Python-coded program was developed to collect data *via* application program interfaces permitted by Twitter and YouTube.

A total of 2,996 tweets were collected containing keywords related to the Hajj and Eid rituals, such as pilgrims: “الحجاج,” Eid: “العيد,” and pilgrimage: “الحج.” Similarly, 1,293 comments were gathered from five videos relating to the Hajj ritual, which were broadcasted on YouTube, including the Arafa sermon: “خطبة عرفة” and the covering of the holy Kaaba: “كسوة الكعبة المشرفة”

### Data analysis

**Figure 1:**
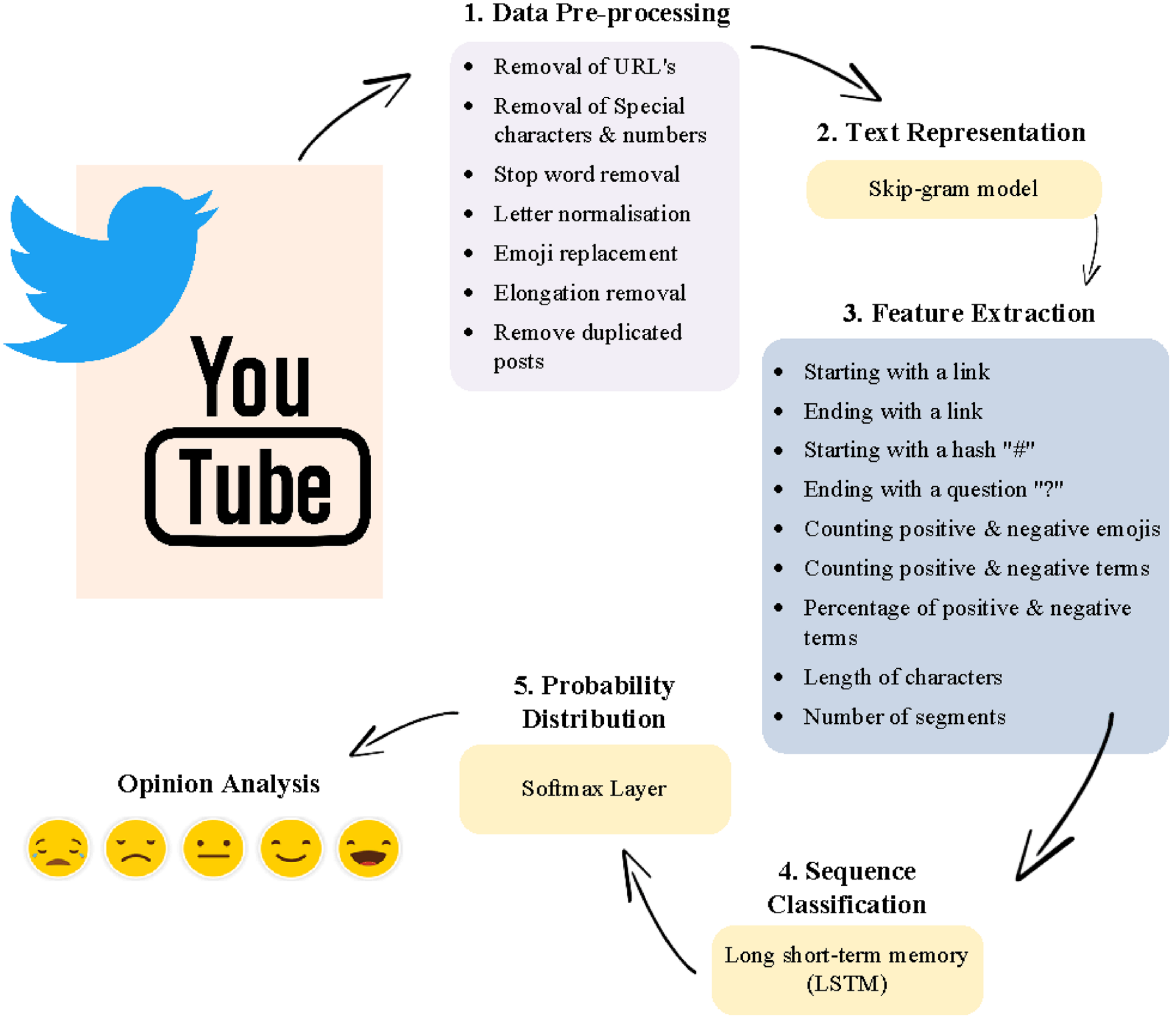
Components of the applied system.

Data analysis involved five main stages, namely, data pre-processing, text representation, feature extraction, sequence classification, and probability distribution, as shown in Fig. 1.

Step 1. Data Preprocessing

Data pre-processing, known as data normalization, is the basis of all SA methods because it consists of a set of procedures aimed at transforming the raw data into a format that can be efficiently processed. The following are the pre-processing steps (Abu Farha & Magdy, 2019):

- Data cleaning: meaningless words and symbols are removed, including URLs, special characters, numbers, duplicated posts, stop words, and diacritics.
- Letter normalization: in this step, the different shapes of some Arabic letters are identified and unified. For example, the letter {ى} is mapped to {ي}, {ة} is mapped to, {ه} and {إ,أ,آ} are mapped to {ا}.
- Emoji replacement: emojis are detected and replaced by specific terms identifying whether the emoji is positive or negative.
- Elongation Removal: repetitive characters and letters are removed, such as {لااا هلااااااا، ياااا} (Pang, Lee & Vaithyanathan, 2002).
- Remove duplicated posts: all posts are verified as non-recurring in the dataset.

Step 2. Text representation

The skip-gram model is employed in the creation of a new word embedding. The texts are represented as a 2D matrix $m \times n$, where $m$ denotes the number of words and $n\;$ is the embedding dimension, which is set to 300 (Abu Farha & Magdy, 2019).

Step 3. Feature extraction and sequence classification

In this step, two deep learning models were combined, the CNN and LSTM models. CNN was used as a feature extractor because it can learn additional features not stated by the embeddings (Abu Farha & Magdy, 2021). Figure 1 provides a set of features extracted by CNN. The LSTM model was used to discover the correlation between extracted features that help express the meaning of aggregated posts. The main goal of combining these models was to extract local features with the help of the convolution layer and use the ordering of those features to learn about the word sequences with the help of the LSTM model (Elzayady, Badran & Salama, 2020). However, CNN-LSTM has drawbacks in terms of parameter selection and data dependency. Choosing the best parameters for deep-learning models remains a significant challenge for researchers (Li, He & Zhang, 2020).

Step 4. Probability distribution

The Softmax function produces the probability distribution of sentiment classes as the final stage in data processing (*i.e*., positive, neutral, and negative).

### Results and Discussion

The results are presented in the form of easy-to-understand tables and graphs. The discussed results reflect the impressions of pilgrims and individuals from within and outside the Makkah community during the 1442 Hajj season, coinciding with the COVID-19 pandemic.

## Evaluation of the cnn-lstm model

### Benchmark datasets

Three of the most popular benchmark datasets were used to evaluate the performance of classification models, including SemEval 2017 Task 4-A (Rosenthal, Farra & Nakov, 2017), and ASTD (Nabil, Aly & Atiya, 2015), and Fake News Challenge (FNS) dataset (Pomerleau & Rao, 2017). The characteristics of SemEval and ASTD datasets are listed in Table 1, which includes the classification of four labels: positive, negative, neutral, and objective. The objective label is given to posts that are categorized as facts rather than opinions. The FNS dataset includes 75,385 tagged instances and 2,587 article bodies, which roughly correspond to 300 headlines as given in Table 2. The classified headlines are labeled as follows: 7.4% of respondents agreed that there is a relationship between an article’s headlines and article bodies; 2% disagreed; 17.7% indicated there is some similarity; and 72.8% argued that the headlines and article bodies are totally different.

**Table 1:** Arabic benchmark datasets.

	SemEval 2017 Task 4-A [42]	ASTD [43]
Positive	2,479	799
Negative	3,492	1,684
Neutral	4,155	832
Objective	–	6,691
Total	10,126	10,006

**Table 2:** Fake news dataset.

Headlines	Tokens	Instances	Agree	Disagree	Discuss	Unrelated
2,587	372	75,385	7.4%	2%	17.7%	72.8%

### Evaluation metrics

The baseline evaluation metrics used to compare the performance of ML approaches in this article are precision, recall (also known as sensitivity), and F-score (Rosenthal, Farra & Nakov, 2017; Baker et al., 2020; Ottom & Nahar, 2021). Noting that, the maximum possible F-score is 1.0, which denotes excellent precision and recall, while the minimum possible F-score is 0, which occurs when either precision or recall are zero. The applied metrics are as follows:

$Accuracy\left( {Acc} \right) = \; \displaystyle{{\left( {TP + TN} \right)} \over {\left( {TP + TN + FP + FN} \right)}}$

$Precision\left( P \right)\; = \displaystyle{{\; TP} \over {\left( {TP + FP} \right)}}\;$

$Recall\left( R \right)\; = \displaystyle{{\; TP} \over {\left( {TP + FN} \right)}}\;$

${F_1} = 2\; \times \displaystyle{{\left( {R \times P} \right)} \over {\left( {R + P} \right)}}$

$F = \displaystyle{{\left( {\; F_1^{C1} + F_1^{C2}} \right)} \over 2},$

where $TP,TN,FP,{\rm and}\; FN$ are true positive, true negative, false positive, and false negative, respectively. ${R^{c1}},{R^{c2}},\; {\rm and\; }{R^{c3\; }}$ denote the recall for positive, negative, and neutral classes, respectively. ${F_1}$ denotes the weighted average of precision and recall.

### Evaluating ML models on SemEval, ASTD and FNS benchmark datasets

The parameter settings used by ML models are given in Table 3. Table 4 illustrates the performance results of several state-of-the-art ML models on the SemEval, ASTD, and FNC datasets. Among the ML models applied on SA datasets are CNN-LSTM (Abu Farha & Magdy, 2019), NileTMRG (Bollen, Mao & Zeng, 2011), Ar-SiTAKA (Jabreel & Moreno, 2017), ELiRF-UPV (González, Pla & Hurtado, 2017), CNN (Heikal, Torki & El-Makky, 2018), LSTM (Heikal, Torki & El-Makky, 2018), Ensemble (CNN/LSTM) (Heikal, Torki & El-Makky, 2018), and Recursive Neural Tensor Networks (RNTN) (Baly et al., 2017). The applied ML models applied on FND dataset are CNN-LSTM (Umer et al., 2020), RoBERTa (Liu et al., 2019) and XLNet (Yang et al., 2019).

**Table 3:** Parameter settings for ML models.

	Arabic sentiment analysis methods				Fake news detection methods		
	CNN-LSTM	CNN	LSTM	Ensemble	CNN-LSTM	RoBERTa	XLNet
LSTM cells	128	**-**	200	200	100	**-**	**-**
Learning rate	0.0001	0.001	0.001	0.001	–	6e−4	4e−4
Dropout	0.2	0.5	0.5	0.5	0.2	0.1	0.1
No. of filters	300	200	–	200	64	–	–
Filter size	3	[3,4,5]	–	[3,4,5]	5	–	–
Pooling size	2	–	–	–	4	–	–
Hidden units	128	30	30	30	100	768	1,024
Number of epochs	–	10	10	10	50	50	–
Batch size	–	50	50	50	32	8k	8,192
Number of layers	–	–	–	–	–	12	24
Attention heads	–	–	–	–	–	12	16

**Table 4:** Experimental results. The best results are in bold.

Datasets	Classification model	${F}$	Acc
SemEval	CNN-LSTM	**0.63**	**0.62**
	NileTMRG	0.61	0.581
	Ar-SiTAKA	0.571	0.563
	ELiRF-UPV	0.467	0.508
ASTD	CNN-LSTM	**0.72**	**0.66**
	CNN	0.641	0.643
	LSTM	0.621	0.648
	Ensemble (CNN/LSTM)	0.645	0.651
	RNTN	0.536	0.585
FNC	CNN-LSTM	**0.978**	**0.978**
	RoBERTa	0.781	0.937
	XLNet	0.760	0.921

The CNN-LSTM model outperformed the competing ML models in all benchmark datasets in terms of classification metrics, including F-score and accuracy. This demonstrates how well CNN-LSTM recognizes and extracts semantic characteristics from text. As a result, the CNN-LSTM model is utilized to conduct more research into pilgrims’ and other people’s perceptions by evaluating their Hajj-related posts on Twitter and YouTube.

## Results and discussion

This section analyzes the impressions of pilgrims and individuals from the Makkah community and beyond on many issues related to the Hajj season 1442 AH through the analysis of posts from social media collected on 1–13 Dhul-Hijjah 1442 AH. A five-point scale was used to express the average SA, with 1 as the lowest value (“significantly dissatisfied/negative”) and 5 as the highest value (“significantly satisfied/positive”).

### Analyzing tweets issued from Mina and Arafa Holy sites

**Figure 2:**
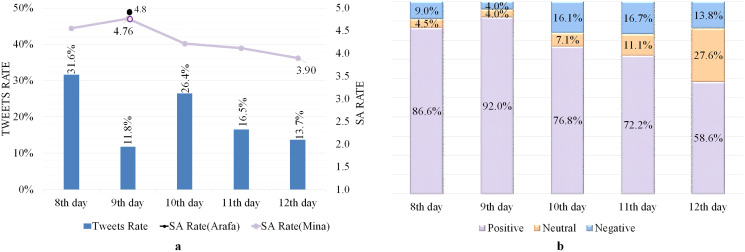
SA of tweets issued from Mina and Arafa Holy sites. (A) Tweets and SA rates. (B) Polarity classification of tweets.

All tweets posted from Mina and Arafa holy sites were collected on 8–12 Dhul-Hijjah because the pilgrims must be present on these days (Shambour & Khan, 2022). Table 5 shows the satisfaction level of the tweeters, with high SA rates of 4.3 and 4.8 out of 5 from within the Mina and Arafa sites, respectively. Figure 2A shows tweets and SA rates during Hajj days, revealing that while tweet rates fluctuate, the SA has high rates ranging from 3.9–4.8, demonstrating that the tweeters have positive moods and emotions. Furthermore, the ninth day has the fewest recorded tweets (11.8%) because it is one of the most important days of Hajj, where pilgrims are preoccupied with worship and supplication, which explains the paucity of tweets gathered.

**Table 5:** SA of tweets issued from Mina and Arafa Holy sites.

Area	No. of tweets (percentage)	SA rate
Mina	213(45%)	4.3
Arafa	259(55%)	4.8

Figure 2B reveals a significant rate of positive tweets on all Hajj days, particularly on Arafa Day (the ninth day), which represented approximately 92% of all tweets and primarily consisted of pleasant supplications and Quranic verses. However, the rate of positive tweets decreases after the ninth day, indicating that some events affected pilgrims’ sentiments during these days.

### Analyzing tweets issued from Makkah, including the keyword “الحجاج” (Pilgrims)

**Figure 3:**
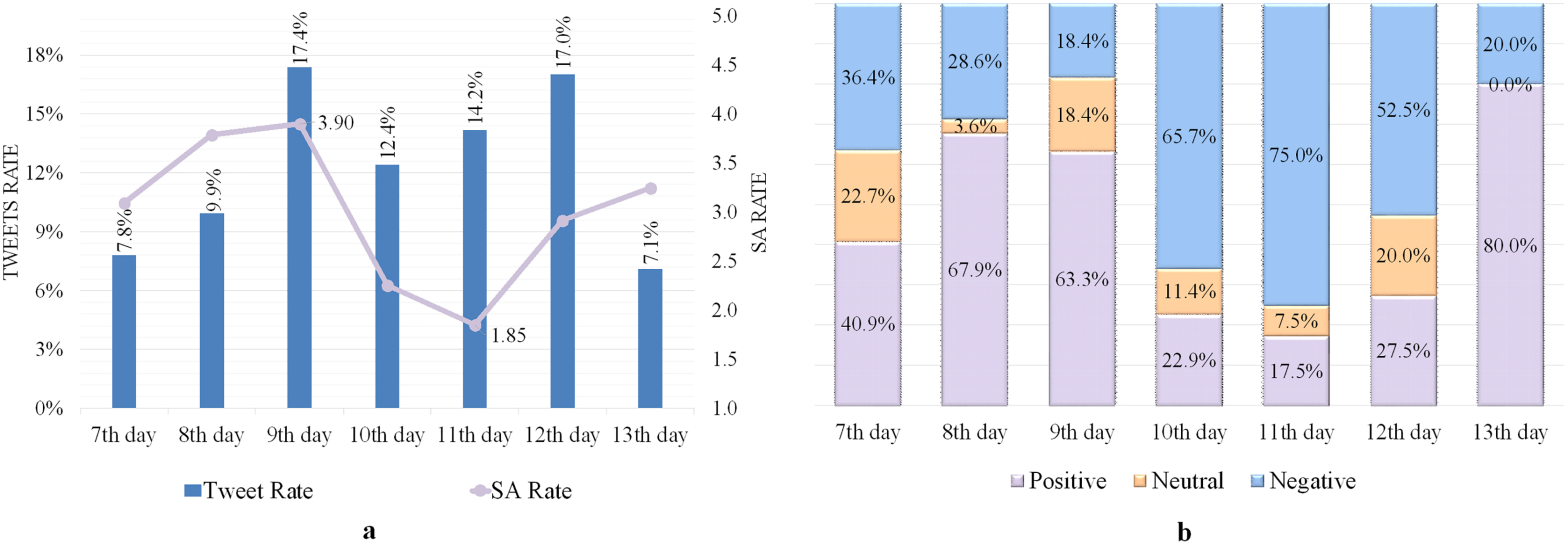
SA of tweets containing the word “الحجاج ” (Pilgrims). (A) Tweets and SA rates. (B) Polarity classification of tweets.

The influence of neutrality on the SA rate was observed on 7–13 Dhul-Hijjah, which was 3.31 out of 5, as indicated in Table 6. The highest SA rate of 3.9 was recorded on the 9th day, indicating a good level of satisfaction, as shown in Fig. 3A. The lowest SA rate of 1.85 was reported on the 11th day, showing a lower level of satisfaction.

**Table 6:** SA of tweets including the keyword "الحجاج" (Pilgrims).

Area	No. of tweets	SA rate
Mecca	242	3.31

Most negative tweets described the nostalgia of Makkah’s people to see their city as crowded with pilgrims as it has been in the Hajj seasons before the COVID-19 pandemic, complaints about the hot weather, and the level of satisfaction for some pilgrims with some services provided, especially for food and transportation.

On the 13th day, the number of positive tweets increased, with the majority praising God Almighty for the fulfillment of the Hajj rituals and the safe return of the pilgrims to their homes. Figure 3B depicts the rate of positive, neutral, and negative tweets on 7–13 Dhul-Hijjah, indicating that positive classified tweets most appeared in the Hajj days, followed by negative and neutral tweets.

### Analyzing tweets issued from Makkah, including the keyword “العيد” (Eid)

Table 7 displays the features of collected tweets from the Makkah community during Islamic festival days (10–13 Dhul-Hijjah), revealing a high positive score of 4.28 out of 5 for tweets using the word “العيد”.

**Table 7:** SA of tweets including the keyword "العيد" (Festival).

Area	No. of tweets	SA rate
Mecca	518	4.28

**Figure 4:**
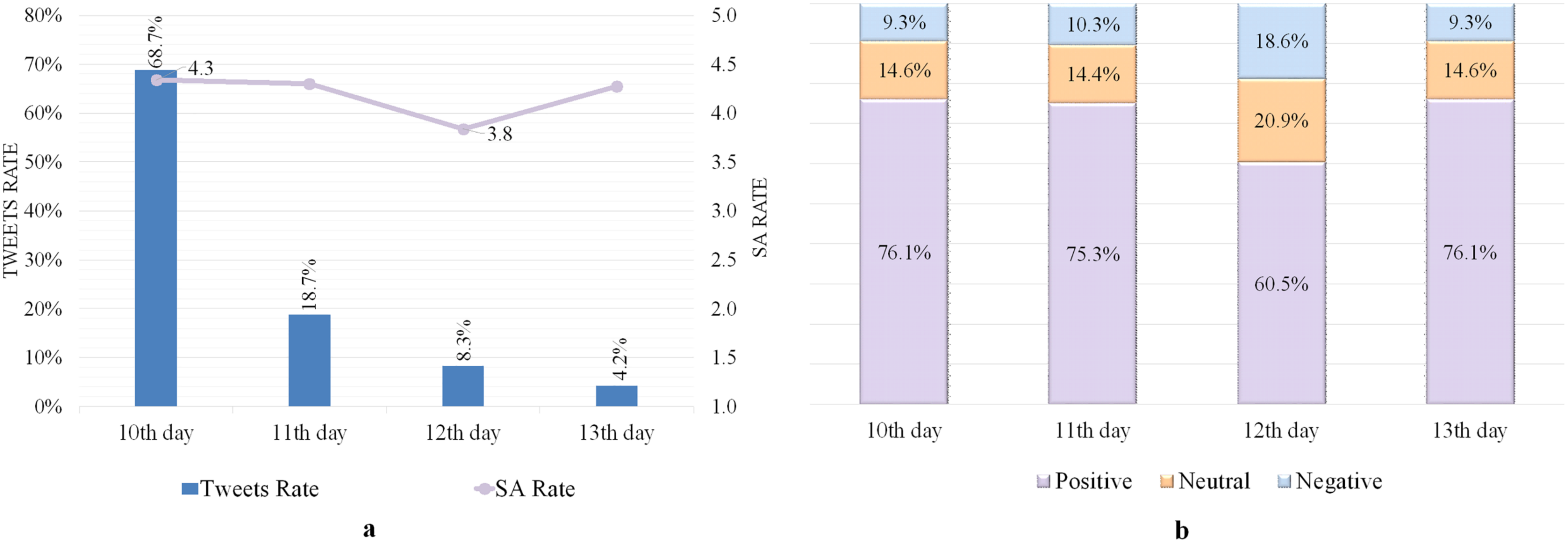
SA of tweets containing the word “العيد” (Eid). (A) Tweets and SA rates. (B) Polarity classification of tweets.

The results in Fig. 4A reveal an apparent happiness influence among tweeters, as evaluated by high level of SA rates, particularly on the first festival day (10th day), when a considerable rate of tweets (68.7%) have a high SA rating of 4.3. The majority of tweets with a positive rating included congratulations and blessings on the occasion of Eid. In contrast, the absence of the Eid celebration activities among Makkah’s people, due to their business serving pilgrims, comprised the most negatively-rating tweets. Figure 4B indicates that positive rated tweets outnumbered negative and neutral rated tweets on all festival days, reaffirming that the festival’s days were filled with pleasant feelings and happiness.

### Analyzing tweets issued from Makkah, including the keyword “الحج” (Pilgrimage)

**Figure 5:**
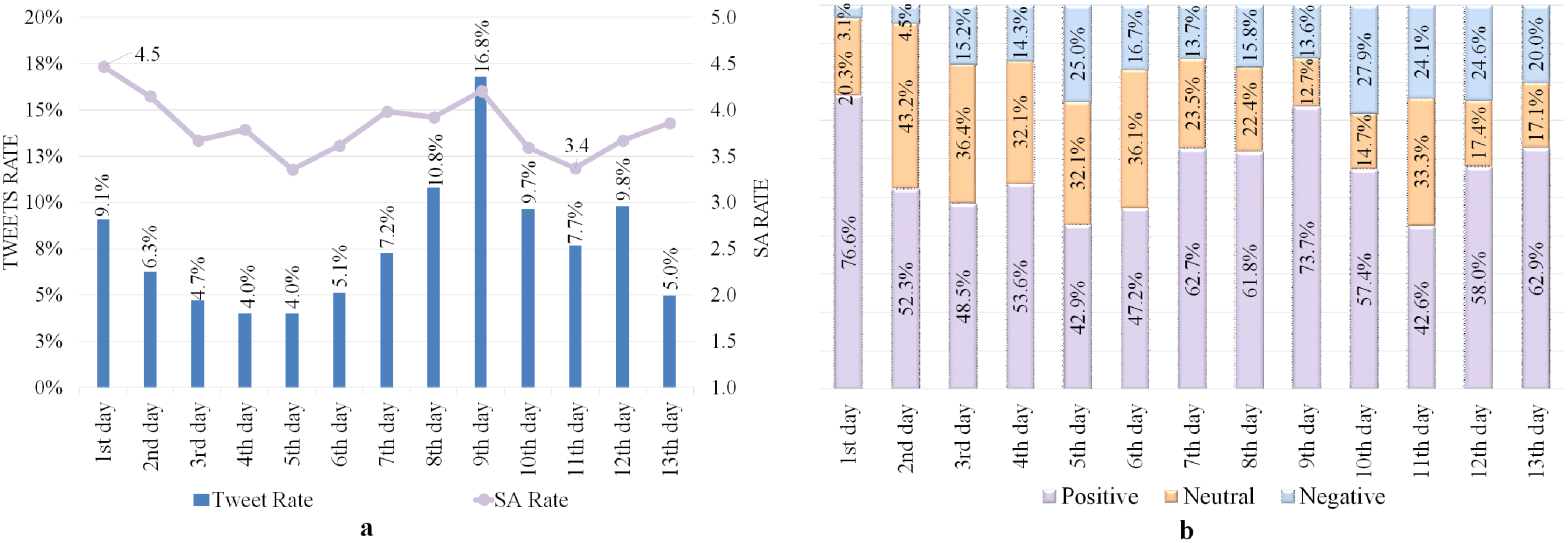
SA of tweets containing the word “الحج ” (Pilgrimage). (A) Tweets and SA rates. (B) Polarity classification of tweets.

As demonstrated in Table 8, 704 tweets were gathered from Makkah on 1–13 Dhul-Hijjah, with an average SA of 3.87, indicating a degree closest to contentment and pleasure. The average SA of the tweeters ranged from above neutral 3.4 to above satisfaction 4.5, as indicated in Fig. 5A. The highest rate of SA was found on the first day of Dhul-Hijjah with 4.5, indicating that the people were quite happy. Furthermore, the results show a decrease in SA rates for the first 5 days of the Hajj and then a progressive increase from the 6th to the 10th day, with the average SA reaching a level exceeding satisfaction on the 10th day, with an SA equal to 4.2. Following that, the SA rate started to decline because of some negatively-rating tweets that reflected the level of pilgrims’ satisfaction for some services they believed were less than their expectations. The SA score returned to an upward trend in the last 2 days (the 12th and 13th); tweets included praise and appreciation to God Almighty as the Hajj rites were performed this year, as well as congratulations and blessings. Although the SA rate changed over the entire period, as shown in Fig. 5B, the positive rated tweets exceeded the neutral and negatively rated tweets, supporting the presence of favorable feelings among Makkah residents toward the Hajj.

**Table 8:** SA of tweets including the keyword “الحج” (Pilgrimage).

Area	No. of tweets	SA rate
Mecca	704	3.87

### Analyzing tweets issued from Makkah, including the keyword “عرفة” (Arafa)

**Figure 6:**
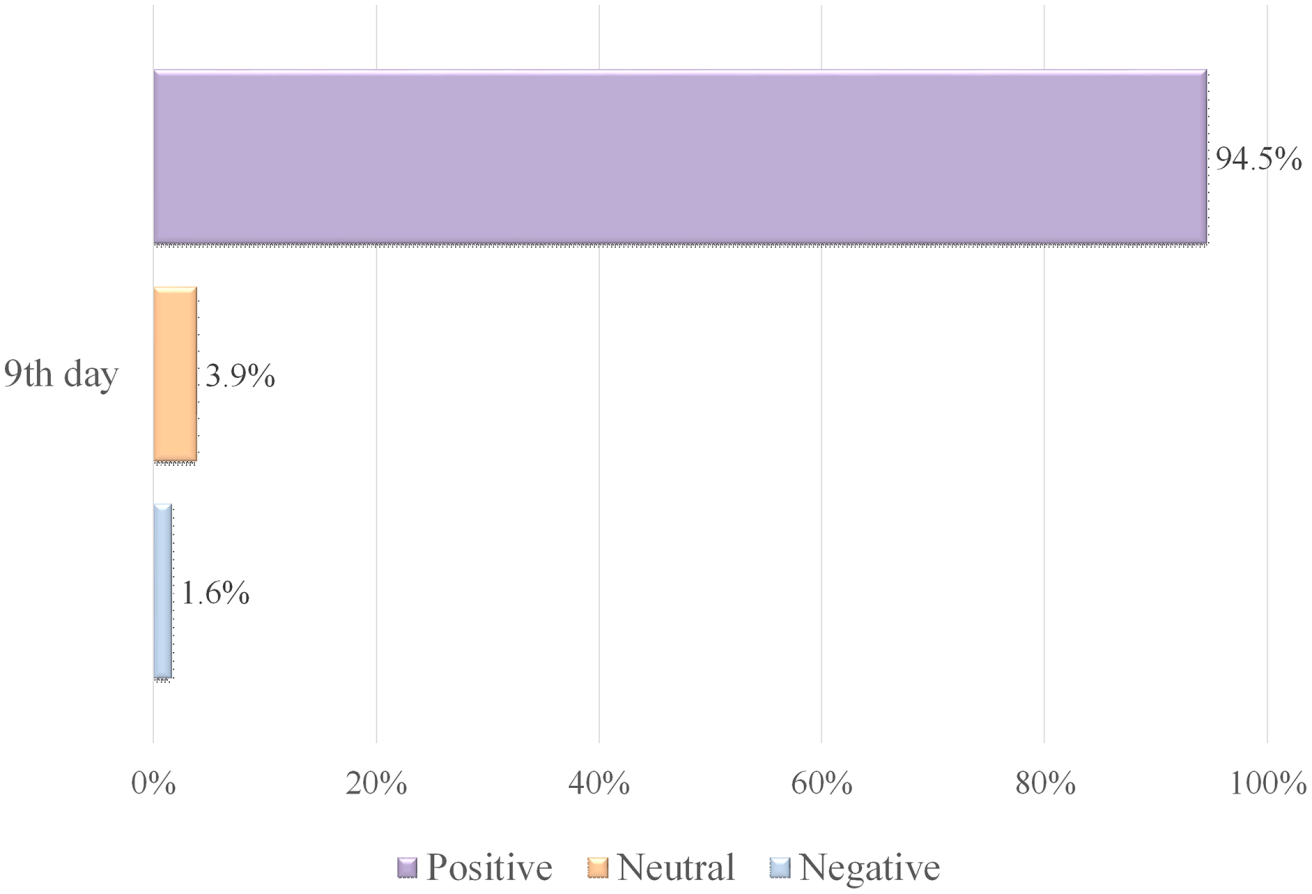
Polarity classification of tweets containing the word “عرفة” (Arafa).

Table 9 illustrates a high level of pleasant emotions and happiness of tweeters in Makkah, with an SA rate of 4.86 for 1,061 collected tweets. Arafa day is the most significant day of Hajj (the ninth day), which requires every pilgrim to be at the Arafa area as the Hajj rituals cannot continue without it. Figure 6 shows that 94.5% of tweets were classified as positive tweets, confirming the prior discussion of having a high amount of pleasant feelings, such as positive supplications and optimistic phrases, whereas only 5.5% were labeled as neutral and negative tweets.

**Table 9:** SA of tweets including the keyword “عرفة” (Arafa).

Area	No. of tweets	SA rate
Arafa	1,061	4.86

### Analyzing comments on YouTube video (Topic: “خطبة عرفة” – “Arafa’s sermon”)

The Saudi Quran, Al Saudiya, and Al-Resalah channels posted a video of the sermon on the day of Arafa on their YouTube channels, garnering 876 comments. Table 10 shows information about the sermon of Arafa posted on the channels, including posted dates, views, likes, dislikes, comments, and SA rates. Results reveal high levels of user satisfaction, with user SA rates reaching 4.17, 4.45, and 4.71 out of 5 for the Saudi Quran, Saudiya, and Al-Resalah channels, respectively, indicating high levels of pleasure with the sermon on Arafa Day.

**Table 10:** YouTube video Information for the sermon of Arafa (“خطبة عرفة”).

	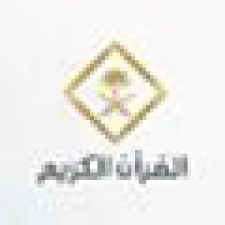 Saudi Quran	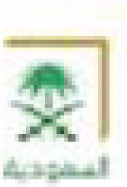 Al Saudiya	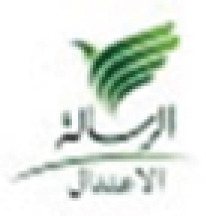 Al-Resalah
Posted date	7/19/2021	7/19/2021	7/19/201
Views	117,371	117,270	139,443
Likes	1,774	2,529	3,658
Dislikes	39	82	127
Comments	172	212	492
SA rate	4.17	4.54	4.71

**Figure 7:**
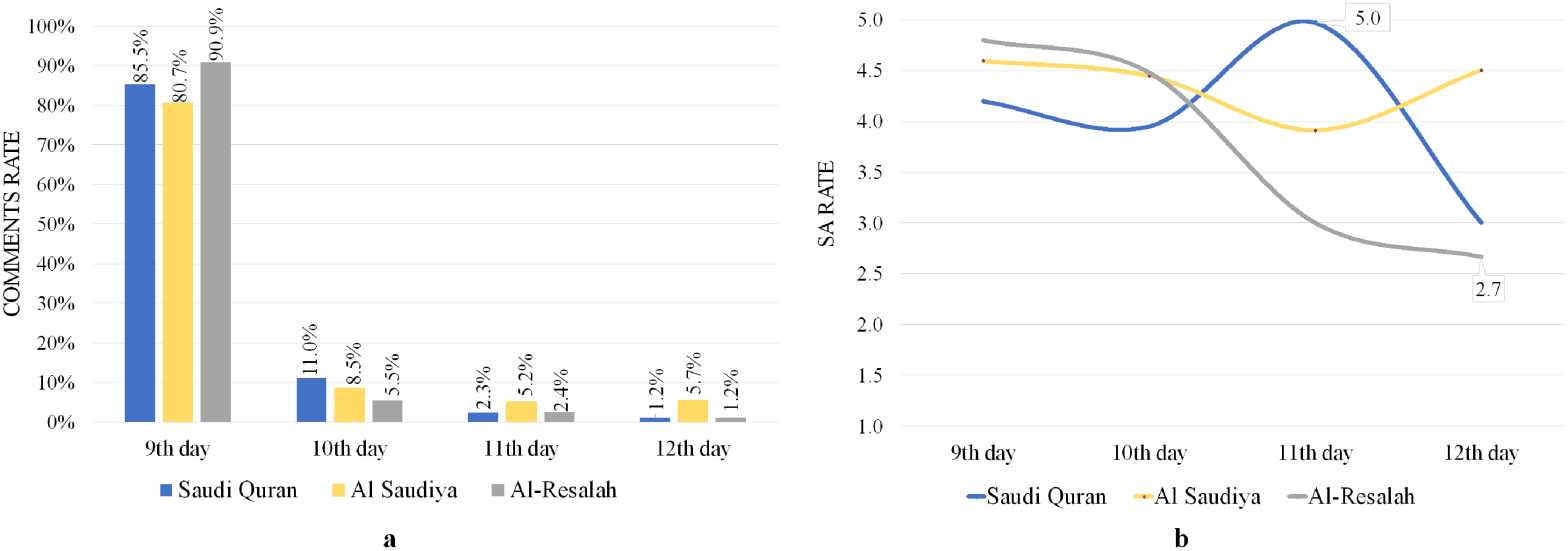
Analyze of YouTube comments on Arafa’s sermon videos. (A) Comments rate. (B) SA rates.

Figure 7A illustrates the average number of daily comments for the three channels, indicating that the majority of comments were made on the same day as the sermon (9 Dhul-Hijjah) and that SA rates were also higher across all channels, as seen in Fig. 7B. The results also show high pleasure for the 11th day of the Saudi Quran channel, with an SA rate of 5.0. These findings demonstrate commentators’ delight, happiness, and eagerness to witness the sermon on Arafa Day. However, as shown in Fig. 7B, the results showed a decline in SA rates after the ninth day, particularly on the 12th day for Saudi Quran and Al-Resalah channels with small percentages of comments 1.2% for each, and 3.0 and 2.7 SA rates, respectively.

### Analyzing comments on YouTube video (Topic: “كسوة الكعبة المشرفة” – “The covering of the Holy Kaaba”)

A total of 417 comments were collected from the Saudi Quran and Al-Arabia channels on YouTube about the video on changing the covering of the Kaaba, which was published on 8 Dhul-Hijjah. Table 11 shows the users’ interaction details and the SA rates. The results in Table 11 reveal high levels of user satisfaction with the Saudi Quran channel’s video, with an SA rate of 4.49, whereas the SA rate of the Al-Arabiya channel’s video was below the level of satisfaction at 3.58.

**Table 11:** YouTube video Information for the covering of the Kaaba (“كسوة الكعبة المشرفة”).

	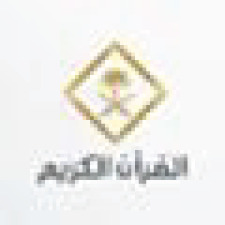 Saudi Quran	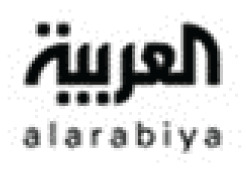 Al-Arabiya
Posted date	7/18/2021	7/18/21
Views	52,623	302,664
Likes	1,405	3,307
Dislikes	20	155
Comments	130	287
SA rate	4.49	3.37

**Figure 8:**
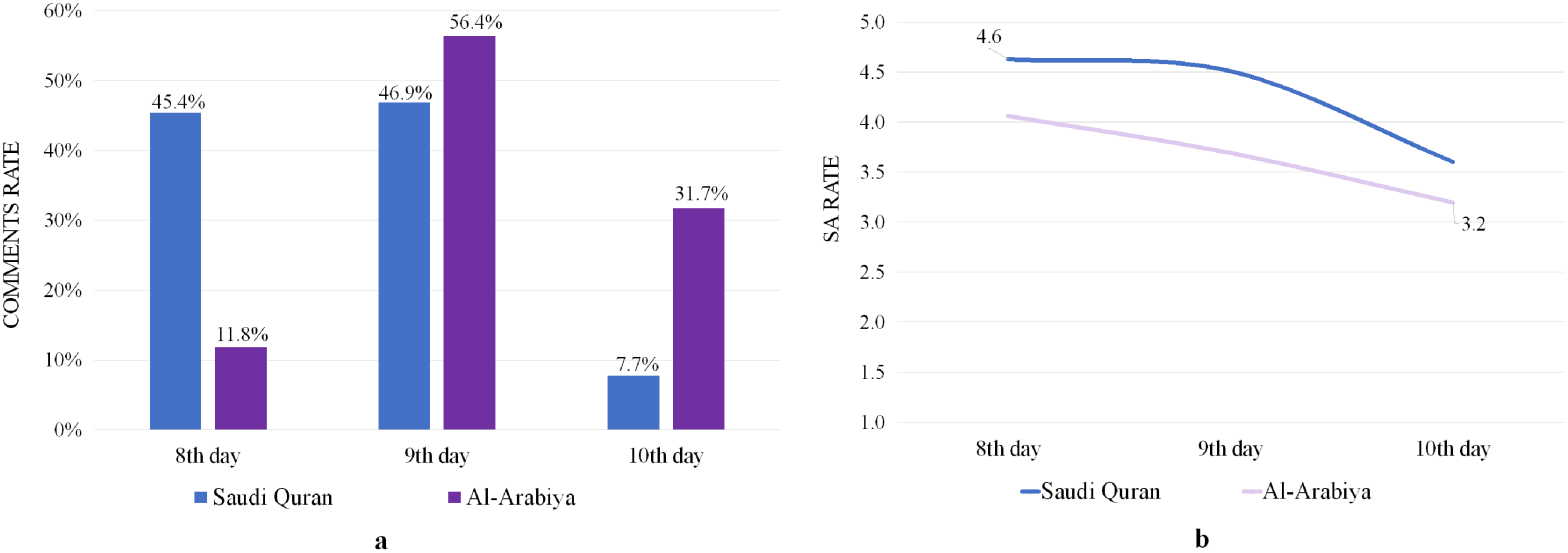
Analyze of YouTube comments on covering of the Holy Kaaba videos. (A) Comments rate. (B) SA rates.

Figure 8A illustrates the average number of daily comments for both channels, revealing that the Saudi Quran and Al-Arabiya channels received the most comments on 9 Dhul-Hijjah, with 46.9% and 56.4%, respectively. The SA rates for the comments recorded on the eighth day were above the level of satisfaction for both channels, as shown in Fig. 8B, but they decreased on the 9th day and continued to decline on the 10th day to a level below satisfaction for both channels. Many types of prayers, praise, and supplications were included in comments with a positive rating, whereas other comments with a negative rating expressed regret over Hajj being limited to a number of pilgrims.

## Conclusion and future work

This study aims to learn about pilgrims’ and others’ impressions from inside and outside the Makkah community in the 1442 Hajj season using a CNN-LSTM deep learning model. The CNN-LSTM model showed better accuracy results on three different benchmark datasets compared to well-known classification models, including NileTMRG, Ar-SiTAKA, ELiRF-UPV, CNN, LSTM, Ensemble (CNN/LSTM), RNTN, RoBERTa and XLNet. More than 4,000 Hajj posts were collected from Twitter and YouTube on 1–12 Dhul-Hijjah 1442 AH. The acquired data were used to expose people’s thoughts and impressions using CNN-LSTM model. The findings revealed several interesting results, including highly positive feelings in tweets collected from Hajj sites and their pleasant experience of being received by residents of Makkah during Hajj days. The negatively-rating tweets reflected the wish of tweeters to host more pilgrims and described the satisfaction level for some services provided to pilgrims. Finally, the SA of YouTube video comments reflected a lot of happiness and optimism among the commenters and eagerness to watch the sermon on Arafa Day.

Future research will look into the performance of other deep learning models, such as ASAD (Hassan et al., 2021) and ArabBert (Antoun, Baly & Hajj, 2020), which were recently presented, on the same dataset and additional benchmark datasets. Future research will also comprehensively compare classification methods in recent literature studies. It will also attempt to understand people’s perspectives on various concerns affected by COVID-19, which has led to changes in many things people had been accustomed to before the pandemic, including e-cooperation, e-education, e-commerce, remote work, virtual work, and e-social networks.

## Supplemental Information

10.7717/peerj-cs.1087/supp-1 Supplemental Information 1 Kiswa dataset. Tweet and Youtube Comments.

## Additional Information and Declarations

### Data Availability

The following information was supplied regarding data availability: The raw data is available in the Supplemental File.

## References

[ref-1] Abdullah M, Hadzikadicy M, Shaikhz S (2019). SEDAT: sentiment and emotion detection in arabic text using CNN-LSTM deep learning.

[ref-2] Abu Farha IA, Magdy W (2019). Mazajak: an online Arabic sentiment analyser.

[ref-3] Abu Farha I, Magdy W (2021). A comparative study of effective approaches for Arabic sentiment analysis. Information Processing & Management.

[ref-4] Alam GN, Sinaga O, Roespinoedji D, Azmi F (2021). The impacts of COVID-19 to Saudi Arabia’s economic sector and Hajj pilgrimage policy of the Kingdom of Saudi Arabia. Turkish Journal of Computer and Mathematics Education.

[ref-5] Alayba A, Palade V, England M, Iqbal R (2017). Arabic language sentiment analysis on health services.

[ref-6] Antoun W, Baly F, Hajj H (2020). AraBERT: transformer-based model for Arabic language understanding.

[ref-7] Baker QB, Shatnawi F, Rawashdeh S, Al-Smadi M, Jararweh Y (2020). Detecting epidemic diseases using sentiment analysis of Arabic tweets. Journal of Universal Computer Science.

[ref-8] Baly R, Badaro G, El-Khoury G, Moukalled R, Aoun R, Hajj H, El-Hajj W, Habash N, Shaban KB (2017). A characterization study of Arabic Twitter data with a benchmarking for state-of-the-art opinion mining models.

[ref-9] Bataineh B, Shambour MK (2019). A robust algorithm for emoji detection in smartphone screenshot images. Journal of ICT Research and Applications.

[ref-10] Bati GFJ (2015). Using big data tools to analyze tweets related to Hajj sentimentally.

[ref-48] Bekkar A, Hssina B, Douzi S, Douzi K (2021). Air-pollution prediction in smart city, deep learning approach. Journal of big Data.

[ref-11] Bollen J, Mao H, Zeng X (2011). Twitter mood predicts the stock market. Journal of Computational Science.

[ref-12] Chaabani Y, Toujani R, Akaichi J (2018). Sentiment analysis method for tracking touristics reviews in social media network. Smart Innovation, Systems and Technologies.

[ref-13] Chen Y, Wang Y, Dong Z, Su J, Han Z, Zhou D, Zhao Y, Bao Y (2021). 2-D regional short-term wind speed forecast based on CNN-LSTM deep learning model. Energy Conversion and Management.

[ref-14] Devlin J, Chang M-W, Lee K, Google KT, Language AI (2019). BERT: pre-training of deep bidirectional transformers for language understanding.

[ref-15] Duwairi R, Hayajneh A, Quwaider M (2021). A deep learning framework for automatic detection of hate speech embedded in Arabic tweets. Arabian Journal for Science and Engineering.

[ref-16] Elgamal M (2016). Sentiment analysis methodology of Twitter data with an application on Hajj season. International Journal of Engineering Research & Science.

[ref-17] Elzayady H, Badran KM, Salama GI (2020). Arabic opinion mining using combined CNN-LSTM models. International Journal of Intelligent Systems and Applications.

[ref-18] Ghourabi A, Mahmood MA, Alzubi QM (2020). A hybrid CNN-LSTM model for SMS spam detection in Arabic and English messages. Future Internet.

[ref-19] Gill H, Khalaf O, Alotaibi Y, Alghamdi Y (2022). Fruit image classification using deep learning. Computers, Materials & Continua.

[ref-20] González J-Á, Pla F, Hurtado L-F (2017). ELiRF-UPV at SemEval-2017 task 4: sentiment analysis using deep learning.

[ref-21] Hassan S, Mubarak H, Abdelali A, Darwish K (2021). ASAD: Arabic social media analytics and understanding.

[ref-22] Heikal M, Torki M, El-Makky N (2018). Sentiment analysis of Arabic Tweets using deep learning. Procedia Computer Science.

[ref-23] Hung BT, Tien LM (2021). Facial expression recognition with CNN-LSTM. Research in Intelligent and Computing in Engineering.

[ref-24] Ilyas MU, Alowibdi JS (2018). Disease tracking in GCC region using Arabic language tweets.

[ref-25] Jabreel M, Moreno A (2017). SiTAKA at SemEval-2017 task 4: sentiment analysis in Twitter based on a rich set of features.

[ref-26] Janjua SH, Siddiqui GF, Sindhu MA, Rashid U (2021). Multi-level aspect based sentiment classification of Twitter data: using hybrid approach in deep learning. Peerj Computer Science.

[ref-27] Jokhdar H, Khan A, Asiri S, Motair W, Assiri A, Alabdulaali M (2021). COVID-19 mitigation plans during Hajj 2020: a success story of zero cases. Health Security.

[ref-28] Li Y, He Y, Zhang M (2020). Prediction of Chinese energy structure based on Convolutional Neural Network-Long Short-Term Memory (CNN-LSTM). Energy Science & Engineering.

[ref-29] Liu Y, Ott M, Goyal N, Du J, Joshi M, Chen D, Levy O, Lewis M, Zettlemoyer L, Stoyanov V (2019). RoBERTa: a robustly optimized BERT pretraining approach (1–13). ArXiv preprint.

[ref-30] Mittal R, Mittal A, Aggarwal I (2021). Identification of affective valence of Twitter generated sentiments during the COVID-19 outbreak. Social Network Analysis and Mining.

[ref-31] Nabil M, Aly M, Atiya A (2015). ASTD: Arabic sentiment tweets dataset.

[ref-32] Ombabi AH, Ouarda W, Alimi AM (2020). Deep learning CNN-LSTM framework for Arabic sentiment analysis using textual information shared in social networks. Social Network Analysis and Mining.

[ref-33] Ottom MA, Nahar KMO (2021). Social media sentiment analysis: the Hajj tweets case study. Journal of Computer Science.

[ref-34] Pang B, Lee L, Vaithyanathan S (2002). Thumbs up? Sentiment classification using machine learning techniques. Proceedings of the 2002 Conference on Empirical Methods in Natural Language Processing (EMNLP 2002).

[ref-35] Pomerleau D, Rao D (2017). Fake news challenge. http://www.fakenewschallenge.org/.

[ref-36] Rosenthal S, Farra N, Nakov P (2017). SemEval-2017 task 4: sentiment analysis in Twitter.

[ref-37] Shambour MKY, Abu-Hashem MA (2021). Analysing lecturers’ perceptions on traditional vs. distance learning: a conceptual study of emergency transferring to distance learning during COVID-19 pandemic. Education and Information Technologies.

[ref-38] Shambour MK, Gutub A (2022). Progress of IoT research technologies and applications serving Hajj and Umrah. Arabian Journal for Science and Engineering.

[ref-39] Shambour MKY, Khan EA (2022). A late acceptance hyper-heuristic approach for the optimization problem of distributing pilgrims over Mina Tents. Journal of Universal Computer Science.

[ref-40] Singh M, Jakhar AK, Pandey S (2021). Sentiment analysis on the impact of coronavirus in social life using the BERT model. Social Network Analysis and Mining.

[ref-41] Statista (2022). • Statista – The statistics portal for market data, market research and market studies. https://www.statista.com/.

[ref-42] Umer M, Imtiaz Z, Ullah S, Mehmood A, Choi G, On B (2020). Fake news stance detection using deep learning architecture (CNN-LSTM). IEEE Access.

[ref-43] Urlam S (2021). Stock market prediction using LSTM and sentiment analysis. Turkish Journal of Computer and Mathematics Education (TURCOMAT).

[ref-44] Vasumathi MT (2021). An effective pomegranate fruit classification based on CNN-LSTM deep learning models. Indian Journal of Science and Technology.

[ref-45] Yang Z, Dai Z, Yang Y, Carbonell J, Salakhutdinov R, Le QV (2019). XLNet: generalized autoregressive pretraining for language understanding.

[ref-46] Zahrani R, Khaldi I, Qahtani K (2015). The impact of understanding social media content on improving performance during the Hajj season, a Twitter case study for the Hajj season 1436.

[ref-47] Zaki UHH, Ibrahim R, Halim SA, Khaidzir KAM, Khaidzir M, Yokoi T (2018). Sentiflood: process model for flood disaster sentiment analysis.

